# Functions of Intrinsically Disordered Regions

**DOI:** 10.3390/biology14070810

**Published:** 2025-07-04

**Authors:** Linhu Xiao, Kun Xia

**Affiliations:** 1 MOE Key Lab of Rare Pediatric Diseases, School of Basic Medicine, Hengyang Medical College, University of South China, Hengyang 421001, China; 2 Institute of Cytology and Genetics, School of Basic Medical Sciences, Hengyang Medical School, University of South China, Hengyang 421001, China

**Keywords:** intrinsically disordered regions, liquid–liquid phase separation, molecular recognition features, post-translation modification

## Abstract

Intrinsically disordered regions (IDRs), defined as protein segments lacking stable tertiary structures, are ubiquitously present in the human proteome and enriched with disease-associated mutations. IDRs harbor molecular recognition features (MoRFs) and post-translational modification sites (e.g., phosphorylation), enabling dynamic intermolecular interactions through conformational plasticity. Furthermore, IDRs drive liquid–liquid phase separation (LLPS) of biomacromolecules via multivalent interactions such as electrostatic attraction and pi–pi interactions, generating biomolecular condensates that are essential throughout the cellular lifecycle. These condensates separate intracellular space, forming a physical barrier to avoid interference between other molecules, thereby improving reaction specificity and efficiency. As a dynamically equilibrated process, LLPS formation and maintenance are regulated by multiple factors, endowing the condensates with rapid responsiveness to environmental cues and functional versatility in modulating diverse signaling cascades. Consequently, disruption of LLPS homeostasis can derail its associated biological processes, ultimately contributing to disease pathogenesis. Moreover, precisely because liquid–liquid phase separation (LLPS) is co-regulated by multiple factors, it may provide novel insights into the pathogenic mechanisms of disorders such as autism spectrum disorder (ASD), which result from the cumulative effects of multiple etiological factors.

## Simple Summary

Intrinsically disordered regions (IDRs) are widely present in the human proteome and can flexibly bind to other biomolecules due to their structural plasticity. At the same time, IDRs provide multivalent weak interactions for liquid–liquid phase separation, which are key factors driving liquid–liquid phase separation. This review focuses on the co-regulation of various factors in liquid–liquid phase separation and systematically summarizes the mechanism and recent advances of diseases caused by the disruption of the dynamic equilibrium of liquid–liquid phase separation when mutations occur on IDRs, aiming to provide valuable insights and references for future research.

## 1. Introduction

### 1.1. IDRs Are Prevalent in the Proteome and Enriched in Disease-Causing Mutations

Intrinsically disordered regions (IDRs) are amino acid sequences lacking stable three-dimensional structures. Their primary structures exhibit enrichment in proline, arginine, glycine, glutamine, serine, lysine, alanine, and glutamic acid residues, frequently organized as repetitive sequences [1,2,3]. Given their absence of fixed conformations, early investigations on protein functionality predominantly focused on structured domains [4], while IDRs were often dismissed as mere flexible linkers devoid of biological significance [5].

However, this conventional paradigm has been reassessed with development in proteomics research. Hyman is the first IDR that has been found to have the biological function of driving LLPS [6]. Analyzing the human proteome using SPOT Disorder, an IDR is defined as ≥30 consecutive amino acids predicted to be disordered. Proteome-wide analyses reveal that over 60% of human proteins contain at least one IDR segment [7,8], with eukaryotes exhibiting significantly higher IDRs content compared to prokaryotes throughout their evolutionary trajectory [9,10].This phylogenetic pattern is substantiated by comparative genomic studies. The conserved expansion of IDRs across eukaryotic lineages correlates with enhanced cellular modularity, functional diversification, and adaptive plasticity—hallmarks of advanced life forms.

A lot of genetic evidence demonstrates that numerous proteins harbor pathogenic mutations in both their IDRs and ordered domains. However, IDRs were previously erroneously considered as merely flexible structural elements with no functional relevance [11]. This misconception led previous studies to predominantly focus on mutations in ordered functional domains, while those in IDRs were either overlooked or annotated as variants of uncertain significance [12], despite mutations on IDRs being disproportionately enriched in disease-associated proteins [3,13]. Notably, up to 25% of disease-related missense mutations are concentrated in IDRs and 20% of disease mutations located in disordered regions cause disorder to order [7,14], definitively disproving the notion that IDRs serve solely as flexible linkers. A growing body of research now establishes that IDRs structurally contribute to diverse biological functions. By discussing the biological functions of IDRs and highlighting their associations with human diseases, this review aims to provide valuable insights and references for future research.

### 1.2. Computational and Experimental Approaches to IDR Detection

The initial characterization of protein structural dynamics and intrinsic disorder relied on biophysical and chemical detection methods. Core experimental approaches included circular dichroism (CD) [15], nuclear magnetic resonance (NMR) [16], dynamic light scattering (DLS) [17], X-ray crystallography [18], single-molecule tracking (SMT) [19], and fluorescence resonance energy transfer (FRET) [20]. Although these methods provide direct validation of IDRs at the amino acid sequence level, they remain labor-intensive and cost-prohibitive for large-scale applications. To address this limitation, computational predictors leveraging sequence features and machine learning models have emerged as efficient tools for proteome-wide IDR screening. The IUPred algorithm [21,22] employs statistical thermodynamics to quantify the free energy of individual residues in disordered states, predicting their propensity to deviate from structured conformations. In contrast, the PONDR framework [9,23] utilizes deep learning algorithms trained on experimentally validated IDR datasets to identify disorder sequences through pattern recognition. Datebase MOBIDB contain structural and functional information about intrinsic protein disorder [24].

### 1.3. Functional Heterogeneity of IDRs: A Length-Based Classification Perspective

**Figure 1:**
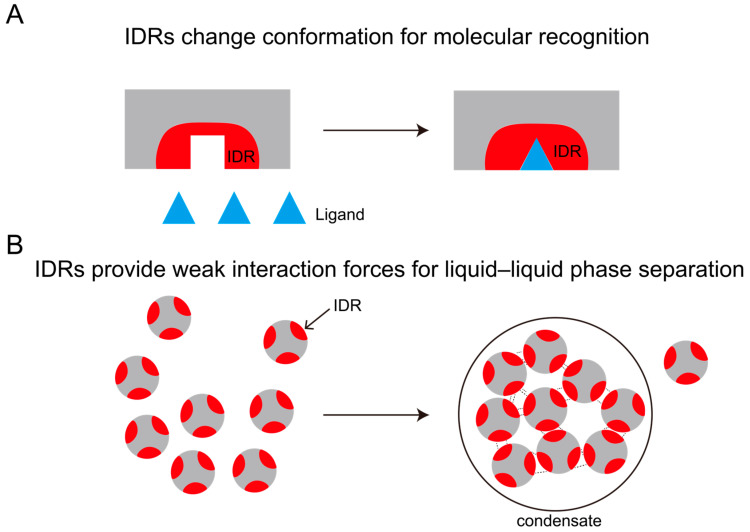
Function of IDRs. (**A**) IDRs recognize molecules through conformational changes; (**B**) IDRs provide weak interaction forces for LLPS.

IDRs can be categorized into two distinct classes based on their length: short IDRs (5–25 residues) [25] and long IDRs (>50 residues) [26,27,28]. Short IDRs frequently harbor molecular recognition elements (MoRFs) and post-translational modification sites. They can recognize molecules by changing their conformation. In contrast, long IDRs predominantly mediate liquid–liquid phase separation (LLPS) of biomacromolecules through multivalent interactions. LLPS refers to the process by which biomacromolecules (such as proteins and RNA) aggregate within cells to form liquid condensates, which become physically segregated from the cytosol. The elevated concentrations of specific proteins and RNA within these condensates enable biochemical reactions to proceed with high specificity and efficiency (Figure 1).

Compared to short IDRs, long IDRs typically exceed 50 residues in length, with their extended sequences enabling the accommodation of repetitive polar/charged motifs (e.g., arginine-rich domains) or hydrophobic modules (e.g., phenylalanine clusters). These structural features facilitate the formation of multivalent interaction networks through electrostatic attractions and pi–pi interactions, thereby significantly enhancing dynamic intermolecular crosslinking capacity [29]. Furthermore, the inherent sequence redundancy of long IDRs confers conformational dynamism, allowing rapid folding–unfolding equilibria that preserve condensate fluidity within phase-separated systems [25]. In contrast, short IDRs (<30 residues) lack sufficient interaction valency to independently drive phase separation, forming only transient aggregates through localized charge complementarity or hydrophobic effects [30]. Their functional contributions to biomolecular condensation primarily depend on cooperative interactions with long IDR scaffolds, serving as anchoring points or regulatory modules within multivalent networks [31,32]. Molecular dynamics simulations further demonstrate that the sequence complexity of long IDRs (≥50 residues) sustains dynamic conformational equilibria essential for stabilizing droplet interfaces, whereas the limited sequence diversity of short IDRs fails to maintain the entropic driving forces required for phase separation [29]. Thus, complexity and multivalent interaction potential establish long IDRs as indispensable structural prerequisites for LLPS.

## 2. Conformational Plasticity of IDRs Enables Molecular Recognition

### 2.1. IDR-Harbored MoRFs with Conformational Flexibility in Molecular Recognition

IDRs frequently harbor MoRFs, which are typically 5–25 residue segments that remain intrinsically disordered in their unbound state but undergo disorder-to-order transitions through induced-fit mechanisms upon target engagement (e.g., proteins, nucleic acids, or small molecules) [33,34]. This phenomenon is most prominently exemplified in biological enzymes. For instance, MoRFs adjacent to phosphorylation sites in the regulatory subunit of cAMP-dependent protein kinase (PKA) interact with cAMP via charge complementarity, triggering structural ordering that releases inhibition of the catalytic subunit and enables downstream protein phosphorylation [35].

The transient ordering of MoRFs confers IDRs with both high specificity and reversibility, allowing rapid environmental responsiveness to signals such as ligand concentration gradients or pH fluctuations. This dynamic behavior positions MoRFs as molecular switches in signal transduction cascades. A paradigm example involves kinase-substrate IDRs where MoRFs mediate dynamic interactions with enzymatic active sites, thereby spatiotemporally regulating phosphorylation events [33,34]. Mutations within IDRs may disrupt their intrinsic disorder [36], impairing their capacity for conformational plasticity and compromising MoRFs [37]. Trans-MoRFs and SHARK can serve as a tool for predicting the structure of MoRFs [38,39].

### 2.2. Post-Translational Modification Sites and Structural Remodeling of Proteins

IDRs are enriched with post-translational modification (PTM) sites—including phosphorylation, acetylation, and ubiquitination modifications—that orchestrate diverse biological functions through distinct biophysical mechanisms. PTMs primarily modulate IDR functionality via three interconnected pathways:

1. Electrostatic Remodeling: Phosphorylation introduces negatively charged phosphate groups, altering the electrostatic landscape of IDRs to regulate partner binding affinities. The C-terminal IDR of p53 exemplifies this mechanism, harboring multiple phosphorylation sites adjacent to DNA-binding motifs. Site-specific phosphorylation patterns (e.g., Ser15 vs. Ser37) enable conformational adaptations that expand p53’s DNA target repertoire, facilitating context-dependent transcriptional regulation [40,41,42]. Similarly, TAU proteins with IDRs as a whole also use phosphorylation modification to change conformation, which wraps microtubules in a flexible conformation under physiological conditions. The modification of multiple phosphorylation sites (such as Ser262, Thr231) can alter the global conformation of TAU protein [43,44].

2. Conformational Restriction: Acetylation and methylation impose steric constraints or stabilize hydrogen-bonding networks that limit IDR flexibility. For example, H3K9 methylation (such as H3K9me3) recruits HP1 protein through hydrophobic interactions and maintains a heterochromatic state [45].

3. Dynamic Structural Regulation: Dynamic regulation of protein structure: The spatiotemporal specificity of PTMs (such as the spatiotemporal distribution of kinase cascade reactions) enables IDRs to integrate upstream signals and achieve fine regulation through dynamic cycles. For example, the IDR of NF—κ B controls its nuclear transport and DNA-binding activity through a phosphorylation–dephosphorylation cycle [43,44].

IDRs are characterized by having the lowest local energy, which makes proteins carrying IDRs possess the characteristic of conformational heterogeneity, and they require less energy to transform between different conformations. Therefore, proteins carrying IDRs are more likely to undergo conformational changes, and have stronger binding ability compared to proteins without IDRs [46,47,48]. Such conformational diversity enables proteins to rapidly respond to changes in environmental factors and present different conformations. This IDR-driven adaptive mechanism provides cells with a real-time response system that bypasses the latency of transcriptional reprogramming, effectively compressing signal-response timelines from hours (gene expression) to seconds (conformational switching) [49].

## 3. IDRs and Liquid–Liquid Phase Separation

### 3.1. IDRs Are Necessary for Liquid–Liquid Phase Separation

LLPS refers to the process by which biomacromolecules (e.g., proteins and RNA) interact to form compositionally distinct liquid droplets within cells [50]. Proteins containing IDRs are uniquely capable of undergoing spontaneous LLPS [7,51,52]. Structural disruptions to IDRs, such as large fragment deletion or missense mutation that may lead to a transition from disorder to order, can abolish LLPS capacity. For instance, hnRNPA1 fails to form condensates in vitro when its C-terminal low-complexity domain (LCD) is disrupted [53]. Conversely, engineered fusion of heterologous IDRs can confer LLPS competence to otherwise non-phase-separating proteins. The RNA-binding protein PTB (polypyrimidine tract-binding protein), which cannot undergo LLPS when mixed with RNA alone, acquires robust phase separation capability upon fusion with IDRs derived from FUS under physiological conditions [54].

The biophysical basis of IDR-driven LLPS lies in their ability to form multivalent interaction networks. The IDRs of these proteins are enriched in a limited number of amino acid types. Among them, charged amino acids—lysine, arginine, glutamate and aspartate—provide electrostatic interactions [55,56,57]. And aromatic amino acids—Phe, Tyr, and Trp—provide pi–pi interactions or cation–pi interactions with other charged amino acid [30,58,59] and arginine-rich motifs facilitate cation–pi interactions [60]. These interconnected weak forces collectively stabilize phase-separated networks, as exemplified by RNA-binding proteins FUS and TDP-43, whose IDRs form dense interaction matrices through synergistic electrostatic and hydrogen-bonding interactions [61]. All of these interaction types—aromatic, polar and charge–charge—are short-lived and provide little structural order to the peptide chain, consistent with the dynamic nature of phase-separated liquids [62].

LLPS typically requires biomolecules to reach a threshold concentration, but IDRs substantially lower this concentration through their multivalent binding capacity. The enhanced avidity from polyvalent interactions allows IDR-containing proteins to form stable droplets at lower concentrations. Furthermore, IDRs can simultaneously engage multiple interaction partners—including RNA and other proteins—thereby promoting co-aggregation of disparate biomolecules and further reducing the concentration threshold for LLPS [63,64,65].

In summary, the intrinsic capacity of IDRs is to drive and regulate LLPS through multivalent interaction networks establishes IDRs as indispensable molecular determinants of phase separation dynamics in cellular systems.

### 3.2. Liquid–Liquid Phase Separation Is Universal in Cells

As previously noted, IDRs are present in approximately 73% of the human proteome [7], suggesting that LLPS driven by IDRs is widely present in cells. Condensates generated by LLPS have the fluidity of the liquid phase. Those condensates undergo a continuous process of assembly, fusion, and dissociation [66,67], while also undergoing changes in composition and subcellular localization [68], exhibiting a high degree of dynamism and maintaining dynamic equilibrium under physiological conditions. The ones that have been thoroughly studied in these condensates are called membraneless organelles, including reproductive granules, stress granules, and nucleoli [6,68,69,70].

#### 3.2.1. LLPS Can Be Found Throughout the Whole Life of Cells

Phase-separated condensates generated through LLPS are ubiquitous throughout the cellular lifecycle, participating in fundamental biological processes ranging from fate determination in undifferentiated cells [6,71,72] and specialized functional maintenance in differentiated cells [73] to the preservation of essential cellular architectures [74]. Substantial evidence establishes LLPS as a critical regulatory mechanism in cellular physiology.

1. LLPS Governs Cell Fate Determination

During germline development in Caenorhabditis elegans, asymmetric distribution of P granules ultimately guides embryonic stem cells to differentiate into germ cells [6]. As germ cells mature, specific components such as ZNFX-1 and WAGO-4 dissociate from P granules to form Z granules, which subsequently regulate RNA translation processes [72].

2. LLPS Underlies Specialized Cellular Functions

In terminally differentiated cells, LLPS remains essential for physiological activities. For instance, phosphorylation at S760 within the IDRs of presynaptic active zone protein LIPRIN-α3 triggers LLPS upon PKC activation. This phase transition recruits RIM and MUNC13 to form functional condensates that regulate neurotransmitter release. Notably, S760 mutation abolishes LIPRIN-α3’s phase separation capacity, resulting in synaptic transmission deficits [73].

3. LLPS Maintains Cellular Structural Integrity

LLPS further plays essential roles in preserving cellular architecture. The nucleolus exemplifies this through its tripartite subcompartment organization, comprising three concentrically arranged regions: the fibrillar center (FC) at the innermost layer, surrounded by the dense fibrillar component (DFC), and outermost granular component (GC) [75]. These subcompartments are formed by LLPS and have different surface tensions due to their different components. This interfacial tension gradient drives the formation of hierarchically nested nucleolar subdomains [74]. The immiscibility between these phase-separated compartments enables functional specialization—FC hosts rDNA transcription machinery, DFC processes rRNA, and GC assembles ribosomal subunits—while preventing molecular crosstalk between adjacent domains [74].

#### 3.2.2. Different Condensates Enrich Different Amino Acids

Biomolecular condensates formed through IDR-driven LLPS are ubiquitous in cellular processes, yet exhibit selectivity toward specific IDR types. Analogous to the hierarchical subcompartmentalization observed in nucleoli, distinct condensates formed by intrinsically disordered proteins (IDPs) with divergent IDR compositions remain mutually immiscible while maintaining spatial coexistence. This selectivity results in characteristic differences in protein composition and concentration across condensates, with such heterogeneity directly underpinning their functional diversification [76]. The amino acid composition of IDRs governs their preferential partitioning into specific condensate types. Proteins harboring IDRs with distinct sequence biases selectively enrich in particular membrane-less organelles. For instance, nucleoli accumulate arginine-enriched IDRs; nuclear speckles concentrate serine/arginine-rich IDRs; and stress granules predominantly incorporate IDRs with small polar residues, supplemented by minor aromatic and charged residues [77]. This sequence-dependent selectivity is exemplified by the HMGB1 frameshift mutation (c.556_559delGAAG; p.(Glu186Argfs*42)), wherein replacement of the native acidic C-terminal IDR with an arginine-rich basic tail alters LLPS behavior. The mutant protein transitions from diffuse nuclear localization to discrete nuclear inclusions, exhibiting an enhanced phase separation capacity that drives aberrant nucleolar partitioning. This mislocalization disrupts nucleolar function, ultimately leading to developmental abnormalities [78]. Notably, even purified proteins from nucleolar subcompartments spontaneously reconstitute immiscible, hierarchically organized condensates in vitro through sequence-encoded LLPS preferences, recapitulating native nucleolar architecture [74].

IDR-mediated LLPS operates throughout the cellular lifecycle as a critical regulator of structural and functional homeostasis. By establishing physical barriers, LLPS isolates biochemical reactions within different condensates, minimizing nonspecific molecular interference while enhancing reaction specificity and efficiency. Sequence-directed partitioning ensures IDPs with distinct amino acid preferences selectively enter specific condensates, where they execute specialized functions. These mutually immiscible yet dynamically interacting compartments coexist in a delicate equilibrium—maintaining structural autonomy while permitting regulated material exchange—thereby collectively sustaining cellular architecture and lifecycle progression.

### 3.3. The Dynamic Equilibrium of Liquid–Liquid Phase Separation Is Co-Regulated by Multiple Factors

LLPS driven by IDRs plays essential roles in diverse cellular processes, making the regulated occurrence of LLPS critical for maintaining normal cellular functions. The paradigm that IDRs serve as primary drivers of LLPS has been experimentally validated in reductionist in vitro systems. However, within the native cellular milieu, cytoplasmic complexity imposes fundamental constraints: endogenous protein concentrations rarely reach the supraphysiological levels required for LLPS induction in vitro, while eukaryotic proteins inherently possess post-translational modifications absent in prokaryotic expression systems. Although LLPS in living cells remains fundamentally driven by weak multivalent interactions mediated by IDRs, its spatiotemporal regulation extends beyond the intrinsic properties of phase-separating proteins. Emerging evidence indicates that LLPS fidelity under physiological conditions is cooperatively governed by multifaceted regulatory mechanisms.

#### 3.3.1. IDRs Are the Critical Factor in LLPS, but Without IDRs Partial Protein Can Still LLPS

The structural integrity of IDRs serves as the determining factor for LLPS, with disease-associated mutations clustered in IDRs frequently disrupting their intrinsic disorder. Notably, five missense mutations—Arg→Trp, Arg→Cys, Glu→Lys, Arg→His, and Arg→Gln—are statistically predominant in impairing IDR functionality [36].

However, emerging studies report that certain proteins devoid of IDRs can undergo phase separation through colocalization with IDR-bearing partners. In zebrafish hematopoietic stem/progenitor cell (HSPC) development, the poly(A)-binding protein CPEB1B—which lacks intrinsic disorder—achieves phase separation by binding to PABPC1B, an IDR-containing protein with prion-like domains (PLDs) [79]. Such dependent phase separators are classified as PS-Part Proteins, contrasting with autonomous PS-Self Proteins. The PhaSePred database systematically catalogs these two categories [80].

Notably, PS-Part Proteins can bypass canonical IDR dependence through mutual interactions. Nuclear proteins NELFA and NELFE each possess short IDRs insufficient for autonomous LLPS. However, combined at specific stoichiometric ratios, their complementary interaction interfaces facilitate cooperative phase separation and condensate formation [81]. This cooperative mechanism expands the functional repertoire of disordered regions.

#### 3.3.2. Protein Concentration Regulation Liquid–Liquid Phase Separation

Protein concentration serves as a critical regulator of LLPS dynamics. During circadian regulation, the core clock protein ATXN2L exhibits sinusoidal oscillations in abundance over 24 h cycles. Concomitant with these concentration fluctuations, ATXN2L-containing condensates assemble during peak expression phases and disassemble as protein levels decline, demonstrating concentration-dependent phase behavior [82]. Furthermore, the bulk cytoplasmic protein concentration modulates LLPS propensity. Supplementation of LLPS buffer with 100 mg/mL bovine serum albumin (BSA) significantly expands the phase separation permissibility window for the RNA-binding protein PTB fused with FUS-derived IDRs [54]. Beyond proteins, nucleic acids and lipids—as fellow biomacromolecules—similarly influence LLPS dynamics through competitive binding or interfacial tension modulation.

#### 3.3.3. Post-Translation Modification Regulates Liquid–Liquid Phase Separation

PTMs is a critical regulating factor of LLPS. For instance, phosphorylation of NELF proteins enhances condensate fluidity and exchange capacity with the surrounding dilute phase [81]. This modification-dependent phase behavior implies that recombinant E. coli-derived proteins—lacking eukaryotic PTMs—may exhibit altered or absent LLPS capacity compared to their native counterparts [81], thereby limiting the biological relevance of in vitro reconstitution approaches for assessing physiological phase separation. Notably, proteins capable of undergoing LLPS exhibit a higher density of PTM sites and elevated modification levels compared to non-phase-separating counterparts, suggesting PTM enrichment as a molecular signature of LLPS competence [83]. Furthermore, IDRs are enriched with PTM sites—including phosphorylation, acetylation, and ubiquitination motifs—that directly modulate both LLPS propensity and conformational transitions through charge state modulation or steric effects [84,85].

#### 3.3.4. Regulation of Liquid–Liquid Phase Separation by Physical or Chemical Factors Such as Salt Ions, pH, and Temperature

The physicochemical properties of LLPS condensates are modulated by ionic species and concentrations. Experimental evidence demonstrates that Mg^2+^ ion concentration positively correlates with both maximum fluorescence intensity post-FRAP (fluorescence recovery after photobleaching) and recovery rate, indicating that Mg^2+^ enhances condensate fluidity and material exchange capacity with the surrounding environment [86]. This liquidity represents the defining characteristic of liquid-phase condensates, as its loss triggers pathological phase transitions such as aggregation or fibrillation—processes frequently implicated in disease pathogenesis [87].

Physical or chemical factors such as pH and temperature can regulate LLPS. Across species, phase separation thresholds exhibit evolutionary adaptation to organismal niches. The critical temperature for Heat shock factor 1 (HSF1) phase separation precisely matches the physiological temperature of its host species, exemplifying this thermal adaptation [88]. Under stress conditions altering ambient pH or temperature, cells initiate protective LLPS responses. Acidic pH shifts and hyperthermia both induce PUB1 oligomerization and subsequent stress granule assembly. While pH normalization permits spontaneous PUB1 condensate dissolution, thermal stress recovery requires chaperone-mediated disassembly via HSP104, showing distinct regulatory mechanisms for environmental stress resolution [89].

**Figure 2:**
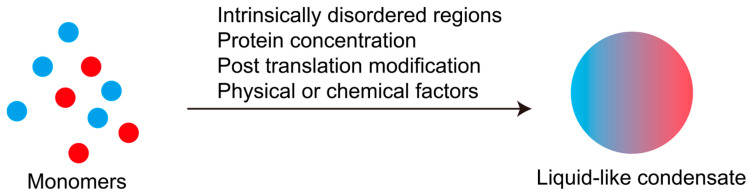
Factors causing liquid–liquid phase separation.

In summary, the homeostatic maintenance of LLPS equilibrium requires coordinated regulation of multiple determinants (Figure 2). This necessitates (1) structural integrity and appropriate post-translational modifications of the IDRs driving phase separation; (2) optimal microenvironmental parameters—including local RNA/protein concentrations, ionic composition, pH, and temperature—within phase-separating compartments. Notably, these regulatory factors dynamically fluctuate during cellular lifecycle progression and adapt to extrinsic environmental challenges. Consequently, LLPS-derived condensates exhibit intrinsic metastability that enables rapid responsiveness to diverse stimuli, positioning them as versatile regulatory hubs for coordinating cellular signaling networks and adaptive responses [90,91,92,93].

### 3.4. The Dynamic Disorder of Liquid–Liquid Phase Separation Is Closely Related to Various Diseases

**Figure 3:**
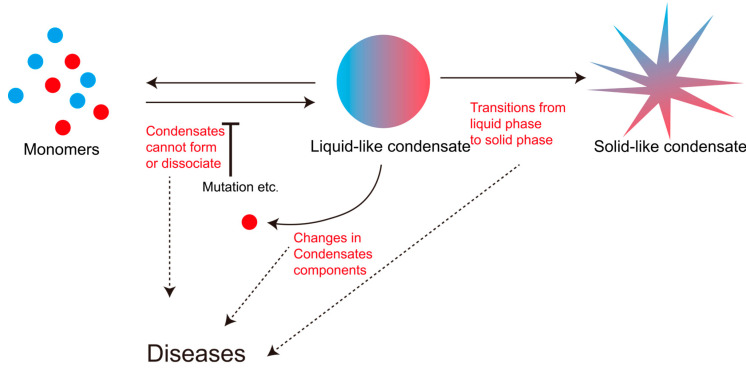
Dysregulation of phase-separation homeostasis leading to diseases.

The biological functions mediated by LLPS condensates are indispensable for cellular physiology. These condensates typically exist in a metastable state, undergoing rapid assembly and disassembly to maintain a delicate dynamic equilibrium within the cellular milieu [68]. This equilibrium—governed by the multifaceted regulatory mechanisms previously discussed—represents a dynamic equilibrium. Common manifestations of LLPS dysregulation include altered protein composition within condensates [32], failed condensate assembly [94], impaired disassembly of mature condensates [87], and pathological liquid-to-solid phase transitions [51,95]. Each form of dynamic disruption can critically compromise cellular homeostasis, ultimately underlying disease pathogenesis (Figure 3).

#### 3.4.1. Changes in Condensates Components

Noonan syndrome, an autosomal dominant disorder, arises from heterozygous missense mutations in the PTPN11 gene in approximately 50% of cases [96]. The PTPN11-encoded non-receptor protein tyrosine phosphatase SHP2 plays a critical role in RAS-MAPK signaling [97]. Wild-type SHP2 remains monomeric under physiological conditions, whereas disease-associated mutations (e.g., D61G, E76A, E76K, Y279C, T468L) identified in Noonan syndrome patients confer aberrant LLPS capacity. These mutant variants spontaneously form condensates that sequester both mutant and wild-type SHP2 [32], creating local concentration gradients that hyperactivate SHP2 phosphatase activity. This gain-of-function mechanism drives constitutive MAPK pathway activation, thereby establishing the molecular pathogenesis of Noonan syndrome.

#### 3.4.2. Condensates Cannot Form

Analysis of FUS-associated pathologies through disease databases such as DisPhaseDB reveals mutation hotspots at residues 229 and 520. Notably, mutations clustered within the 300-residue segment correlate with disease pathogenesis—a region computationally predicted by phase separation propensity tools FuzDrop and PSPHunter to harbor critical LLPS-determining residues [94]. The Lys312Gln (K312Q) mutation within this domain, which is clinically associated with ductal breast carcinoma [98], significantly impairs FUS phase separation capacity in vitro. Mechanistically, this substitution disrupts pi–cation interactions between aromatic and charged residues. Replaced residues normally have the ability to stabilize LLPS-competent conformations.

#### 3.4.3. Condensates Cannot Dissociate

FOXM1, an oncoprotein capable of undergoing LLPS, forms nuclear condensates that drive oncogene transcription and promote metastasis in triple-negative breast cancer (TNBC). The S376 residue within FOXM1 serves as a phosphorylation target for AMP-activated protein kinase (AMPK), which enhances phosphorylation of its IDRs. In cancer patients harboring the S376A mutation, FOXM1 cannot undergo phosphorylation at this residue. Compared to phosphorylated FOXM1, the non-phosphorylated S376A mutant exhibits enhanced LLPS capacity and transcriptional activity. Reduced phosphorylation stabilizes FOXM1 condensates, leading to persistent hyperactivation of proto-oncogene transcription programs that fuel tumor progression and metastatic dissemination [87]. Therapeutic strategies targeting LLPS dysregulation are emerging. In the FOXM1 paradigm where hypophosphorylated IDRs stabilize oncogenic condensates, the disruptive peptide FIP4 successfully inhibits tumor progression by dissolving phase-separated FOXM1 condensates [87]. Those kinds of treatments that involve dissolving condensates are very common. A small-molecule EGCG from green tea can be used to disaggregate tau and other amyloid fibrils [57].

#### 3.4.4. Condensates Transitions from Liquid Phase to Solid Phase

The transition from liquid–liquid to liquid–solid phase separation represents a central pathological mechanism across multiple diseases, which is particularly prevalent in neurodevelopmental and neurodegenerative disorders (Table 1).

In amyotrophic lateral sclerosis (ALS), glycine and proline residues maintain FUS protein liquidity, while serine and glutamine residues promote its liquid–solid transition [58,99,100]. The G561E mutation within FUS’s IDRs induces structural reorganization from disordered conformations to radially distributed fibrillar aggregates. This phase transition converts liquid FUS condensates into solid-state assemblies [51], triggering persistent proteostatic stress through aberrant protein aggregation in neurons.

Neurodegenerative diseases exhibit analogous pathological transitions [101]. The microtubule-associated protein Tau undergoes physiological LLPS in neuronal regions requiring microtubule nucleation, where it transiently stores Tau monomers to regulate microtubule dynamics [24,95]. However, IDR mutations or pathological phosphorylation drive Tau condensates through sequential phase transitions—from liquid droplets to gel-like states and, ultimately, insoluble fibrillar aggregates. While in vitro studies recapitulate this transition through prolonged protein incubation, in vivo propagation occurs via trans-neuronal spreading of pathological conformers [102]. Tau fibrillization constitutes a hallmark of Alzheimer’s disease and related tauopathies [103].

**Table 1:** Diseases and clinical manifestations caused by liquid–solid phase separation.

Disease	Solid Phase Protein	Clinical Manifestation
Alzheimer’s	Tau	Loss of cognitive functioning and behavioral abilities
Parkinson’s [104]	α-synuclein	Uncontrollable shaking and difficulties with balance and coordination
ALS	TDP-43, FUS	Twitching and cramping of muscles; trouble breathing; trouble swallowing; paralysis
Huntington’s [105]	mHTT	Strange and uncontrolled movements; loss of memory and judgment

In summary, LLPS of biomacromolecules involves dynamic equilibrium between monomeric and condensed states, governed by multifactorial regulation: local protein concentration gradients, the physicochemical microenvironment (ionic strength, pH, temperature), ligand/chaperone availability, post-translational modification status, and mutations on IDRs’ profiles. Perturbations in these parameters can either initiate pathological LLPS or destabilize existing condensates, destroying the dynamic equilibrium.

## 4. Conclusions

IDRs are ubiquitously present in the human proteome [25], where their structural plasticity enables molecular recognition through conformational switching [29]. Simultaneously, IDRs provide multivalent weak interactions that drive and regulate LLPS, establishing them as critical architectural elements in phase separation dynamics [50].

MoRFs embedded within IDRs undergo post-translational modification-induced conformational changes, enabling dynamic nucleic acid and protein binding [30,31,32]. This rapid conformational adaptability enhances cellular responsiveness to environmental fluctuations [27,28].

The dynamic nature of LLPS allows condensates to function as sensitive signaling hubs that coordinate diverse cellular processes. Membraneless organelles formed through LLPS play essential roles in fundamental biological activities including DNA repair [106], RNA transcription [107], protein translation, and post-translational modification. By compartmentalizing biochemical reactions, LLPS ensures efficient and non-interfering execution of parallel cellular programs [108]. Conversely, disruption of LLPS homeostatic equilibrium compromises these functions, ultimately contributing to disease pathogenesis.

Notably, 21.7% of disease-associated missense mutations are enriched in IDRs, with ≥20% inducing local disorder-to-order transitions and others disrupting IDR-mediated functions through conformational destabilization [36]. This mutation clustering is particularly pronounced in autism spectrum disorder (ASD), a neurodevelopmental condition with 50–90% heritability [109,110]. ASD pathogenesis involves cumulative deleterious variants across >100 high-confidence risk genes [111,112], many encoding IDR-containing proteins with predicted LLPS propensity. Genetic analyses reveal 69% of ASD-associated mutations localize to IDRs on average [7], affecting processes spanning chromatin remodeling, mRNA processing, synaptic function, and receptor signaling [108]. Progressive accumulation of LLPS-perturbing mutations eventually exceeds critical thresholds for condensate stability, providing a mechanistic framework for ASD’s polygenic etiology.

## Data Availability

No new data were created or analyzed in this study.

## Abbreviations

IDRs	Intrinsically disordered regions.
LLPS	Liquid–liquid phase separation.
MoRFs	Molecular recognition features.
CD	Circular dichroism.
NMR	Nuclear magnetic resonance.
DLS	Dynamic light scattering.
SMT	Single-molecule tracking.
FRET	Fluorescence resonance energy transfer.
PTM	Post-translational modification.
LCD	Low-complexity domain.
FC	Fibrillar center.
DFC	Dense fibrillar component.
GC	Granular component.
IDPs	Intrinsically disordered proteins.
HSPC	Hematopoietic stem/progenitor cell.
PLDs	Prion-like domains.
FRAP	Fluorescence recovery after photobleaching.
HSF1	Heat shock factor 1.
ALS	Amyotrophic lateral sclerosis.
TNBC	Triple-negative breast cancer.
ASD	Autism spectrum disorder.
